# Long-term outcomes in Ornithine Transcarbamylase deficiency: a series of 90 patients

**DOI:** 10.1186/s13023-015-0266-1

**Published:** 2015-05-10

**Authors:** Anais Brassier, Stephanie Gobin, Jean Baptiste Arnoux, Vassili Valayannopoulos, Florence Habarou, Manoelle Kossorotoff, Aude Servais, Valerie Barbier, Sandrine Dubois, Guy Touati, Robert Barouki, Fabrice Lesage, Laurent Dupic, Jean Paul Bonnefont, Chris Ottolenghi, Pascale De Lonlay

**Affiliations:** Reference Center of Inherited Metabolic Diseases and units of metabolism and neurology, 149 rue de Sèvres, 75015 Paris, France; Service de Biochimie Métabolique, Paris, France; Service de Génétique, Paris, France; Service de Réanimation pédiatrique, Paris, France; Université Paris Descartes, Institut Imagine, Hôpital Necker-Enfants Malades, APHP, Paris, France

**Keywords:** Urea Cycle Disorders (UCD), Ornithine Transcarbamylase deficiency (OTCD), Hyperammonemia, OTC gene mutations, Hyperammonemic encephalopathy, Acute metabolic decompensation, Hemodialysis, Liver transplantation

## Abstract

**Background:**

The principal aim of this study was to investigate the long-term outcomes of a large cohort of patients with ornithine transcarbamylase deficiency (OTCD) who were followed up at a single medical center.

**Methods:**

We analyzed clinical, biochemical and genetic parameters of 90 patients (84 families, 48 males and 42 females) with OTCD between 1971 and 2011.

**Results:**

Twenty-seven patients (22 boys, 5 girls) had a neonatal presentation; 52 patients had an “intermediate” late-onset form of the disease (21 boys, 31 girls) that was revealed between 1 month and 16 years; and 11 patients (5 boys, 6 girls) presented in adulthood (16 to 55 years). Patients with a neonatal presentation had increased mortality (90% versus 13% in late-onset forms) and peak plasma ammonium (mean value: 960 μmol/L versus 500 μmol/L) and glutamine (mean value: 4110 μmol/L versus 1000 μmol/L) levels at diagnosis. All of the neonatal forms displayed a greater number of acute decompensations (mean value: 6.2/patient versus 2.5 and 1.4 in infants and adults, respectively). In the adult group, some patients even recently died at the time of presentation during their first episode of coma. Molecular analyses identified a deleterious mutation in 59/68 patients investigated. Single base substitutions were detected more frequently than deletions (69% and 12%, respectively), with a recurrent mutation identified in the late-onset groups (pArg40 His; 13% in infants, 57% in adults); inherited mutations represented half of the cases. The neurological score did not differ significantly between the patients who were alive in the neonatal or late-onset groups and did not correlate with the peak ammonia and plasma glutamine concentrations at diagnosis. However, in late-onset forms of the disease, ammonia levels adjusted according to the glutamine/citrulline ratio at diagnosis were borderline predictors of low IQ (p = 0.12 by logistic regression; area under the receiver operating characteristic curve of 76%, p <0.05).

**Conclusions:**

OTCD remains a severe disease, even in adult-onset patients for whom the prevention of metabolic decompensations is crucial. The combination of biochemical markers warrants further investigations to provide additional prognostic information regarding the neurological outcomes of patients with OTCD.

## Introduction

Ornithine transcarbamylase deficiency (OTCD), an X-linked disorder, is the most common urea cycle disorder (UCD) with an incidence between 1/17 000 in the USA [1] and 1/60 000 in Finland [2], whereas the overall frequency of urea cycle disorders is approximately 1 in 8000 newborns [3]. The gene encoding OTC is located on Xp21.1, and the enzyme is expressed in the liver and gut. OTC is a mitochondrial enzyme that catalyzes the conversion of ornithine and carbamoyl phosphate to citrulline.

The clinical presentation of OTCD is very heterogeneous. Affected hemizygous males usually present in the neonatal period with acute hyperammonemic coma due to a severe enzymatic deficiency, whereas the clinical severity of heterozygous affected females varies considerably, depending partially on the pattern of inactivation of the X chromosome. When the enzymatic deficiency is partial, the onset of symptoms can be delayed to childhood or adult age for males and females. Hepatodigestive, neurological or psychiatric symptoms can be present. Biochemically, OTCD impairs the synthesis of citrulline and urea, which leads to the accumulation of ammonium, glutamine, and other amino acids, contrasting with the low levels of citrulline and diversion of carbamoylphosphate into pyrimidine synthesis and leading to an increase in the excretion of urinary orotic acid [4]. Until the early 2000s, diagnosis was confirmed by the measurement of liver or gut OTC activity, followed mainly by genetic mutation analysis of the OTC gene, which is considered the reference test.

The principles of OTCD treatment are dietary protein restriction, arginine and citrulline supplementation, and the induction of nitrogen excretion via the administration of sodium phenylbutyrate and/or sodium benzoate [5-7]. Liver transplantation is an alternative to medical therapy for severe OTCD in the neonatal form, or in the case of frequent episodes of recurrent hyperammonemia or poor metabolic status [8,9].

Relatively little is known about the neurological and metabolic evolution of treated patients with OTCD.

To better delineate the long-term outcome of patients with OTCD, we analyzed clinical, biochemical, therapeutic, and genetic parameters as well as the outcomes of a large cohort of patients who were reviewed over 40 years.

## Methods

### Patients

Between 1971 and 2011, 90 patients (84 families, 48 males and 42 females) were diagnosed and followed up for OTCD at Necker Enfants-Malades Hospital. The patients were born between 1939 and 2011.

The inclusion criteria were that each patient had been investigated in the same unit at Necker hospital and that heterozygous females were symptomatic and had a confirmed OTC mutation or OTC enzymatic deficiency. Patients with no confirmed enzymatic or molecular diagnosis were excluded, as were those who were diagnosed at Necker Enfants-Malades hospital but followed-up in another hospital.

We reviewed the neonatal presentations (0-30 days) and late-onset phenotypes (after 30 days) separately. Regarding the late-onset group, we distinguished patients who were diagnosed between 30 days and 16 years of age (referred to as group 1 mth-16 y) from those who were diagnosed after 16 years of age (referred to as group >16 y).

In addition, asymptomatic carriers (relatives of patients) were studied at the molecular level.

The medical data were retrospectively obtained from medical reports, including physical examinations, neuropsychological testing, nutritional parameters and drug treatments. We recorded each hyperammonemic episode and biochemical parameter at diagnosis and during the follow up. We correlated the clinical presentation and patient follow up to the biochemical and/or genetic data for each patient.

This work was approved by our institutional ethical committee after declaration to the *Département de la Recherche Clinique et du Développement*, and informed consent was obtained from the parents.

### Clinical data

Clinical symptoms at diagnosis were classified into three different phenotypes: neurological, hepatic, and psychiatric phenotypes.

### Biochemical parameters

Multiple biochemical parameters were measured at diagnosis, including peak ammonemia levels (N < 50 μmol/L), plasma amino acids with glutamine (N: 530 +/- 81 μmol/L), arginine (79 +/-25 μmol/L) and citrulline (N: 26+/-8 μmol/L), and urinary excretion of orotic acid (N < 6 μmol/mmol of creatinine).

### Enzymatic and molecular diagnosis

The diagnosis was confirmed by measurement of the hepatic or intestinal OTC enzymatic activity [10] or by mutation analysis of the OTC gene by direct sequencing of the 10 exons [11]. In cases in which no mutation was identified by sequencing analysis, multiplex ligation-dependent probe amplification (MLPA) (SALSA MLPA P079 OTC kit, MRC-Holland, Amsterdam, The Netherlands) was performed according to the manufacturer’s instructions [12,13].

### Therapeutic data

The medical treatment consisted of a low-protein diet that was adapted to the tolerance of the individual combined with arginine and/or citrulline supplementation and sodium phenylbutyrate and/or sodium benzoate administration, depending on the onset and severity of the disease. Nocturnal enteral feeding was used for patients with oral difficulties. Emergency treatment with high-caloric intake providing glucose and lipids was performed during catabolic states. Concerning neonatal management, our practices improved over time through the use of more effective hemofiltration since 2000, the combination of intravenous sodium phenylacetate and sodium benzoate [7] and the systematic association of arginine, citrulline, sodium benzoate and sodium phenylbutyrate.

### Follow up: acute metabolic decompensations and biochemical markers

The number of metabolic decompensations with clinical symptoms and plasma ammonia concentration greater than 100 μmol/L and/or hepatic failure requiring emergency treatment and hospitalization were used to classify the patients into three groups. Group 0 included none or one metabolic decompensation during the follow-up period. Group 1 included 2 to 10 episodes of decompensation during follow-up and group 2 included at least 11 decompensations during follow-up.

The biochemical markers measured during follow-up excluded those corresponding to acute metabolic decompensations.

### Neurological outcome

The psychomotor evaluation was performed using standardized psychological tests according to the age of the patient: the developmental quotient (DQ) by the Brunet-Lezine test; the intellectual quotient (IQ) by WPPSI-R and WPPSI-III (Wechsler Preschool and Primary Scale of Intelligence); WISC-III and WISC-IV (Wechsler Intelligence Scale for Children). Two others tests were used in a subset of cases: NNAT (Naglieri Non-Verbal Ability Test) and NEMI (New Intelligence Metrical Scale).

The other criteria used to assess the neurological outcome were the schooling of the patient, which was classified as normal with normal academic achievement for age, in remedial classes or institutional education if the patient had a minor handicap, and no schooling in the presence of severe mental retardation. For adults, we investigated socio-professional integration and autonomy.

We established a neurological score based on the following parameters: intellectual performance adapted to age, academic achievement and motor disorder with limited autonomy. Patients were classified into group 1 if they had an estimated IQ > 80 with normal academic achievement, group 2 if they had moderate mental retardation (50 < IQ < 80) and neurological sequelae leading to institutional education, and group 3 if they had severe mental retardation (IQ < 50) and neurological damage with no motor autonomy.

### Statistical analysis

The results are expressed as the mean and median values (with minimum-maximum range values) for quantitative data and as percents for categorical data. Comparisons of categorical data were performed using Fisher’s exact test. Overall survival was calculated from birth to last follow-up or death and was estimated using the Kaplan-Meier method. Statistical significance was set at p < 0.05. Survival, area under the curve, and linear and logistic regression analyses were performed with survfit, pROC and glm modules in R (http://cran.r-project.org/).

## Results

Among the 90 patients with OTCD, 53% (48 patients) were males and 47% (42 patients) were heterozygous symptomatic females. Twenty-seven patients (30%) were diagnosed with a neonatal presentation, including 22 males (81%) and 5 girls (19%). The median age at diagnosis was 2 days, after the exclusion of 6 asymptomatic patients who were treated immediately after birth due to a prenatal diagnosis that followed an index case in the corresponding families. Fifty-two patients (58%) had a late-onset form of the disease that was revealed between 1 month and 16 years (median age at diagnosis: 1.3 years), including 31 girls (60%) and 21 males (40%). Eleven patients (11%) presented disease in adulthood and adolescence after 16 years of age (median age at diagnosis: 28 years), including 5 males and 6 girls (cf Table 1).

**Table 1:** **Epidemiological characteristics of the patients**

	**Neonates**	**Group 1 m-16y**	**Group >16y**
Number of cases	27	52	11
Males	22	21	5
females	5	31	6
Number of deceased	20	7	1
at diagnosis	13	5	1
during follow up	7	2	0
Number of decompensations per patient (and relative to mean length of follow-up)*	6,2 (1.0/yr)	2,5 (1/10 yrs)	1,4 (<1/20 yrs)
Neurological score (IQ) at last follow up, N > 80	90	92	Normal socio-professional insertion
Mean peak plasma ammonia at diagnosis (μmol/L), N < 50 μmol/L	960	500	
Mean plasma glutamine at diagnosis (μmol/L), N: 530 +/- 81 μmol/L	4110	1000	
Plasma citrulline at diagnosis (μmol/L), N: 26+/-8 μmol/L	5	15	14

*Only the patients that survived beyond neonatal life were included.

### Mortality

**Figure 1:**
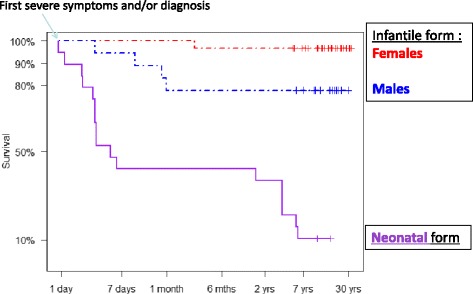
Survival of OTCD patients by age of onset. Comparison of survival between the 1 mth-16 y and neonatal group. In the 1 mth-16 y group, the “critical” period (risk of death) is that between the first severe symptoms and diagnosis. In the neonatal form, there are two high-risk age intervals: the first days of life and the period between 1 and 7 years of age.

**Figure 2:**
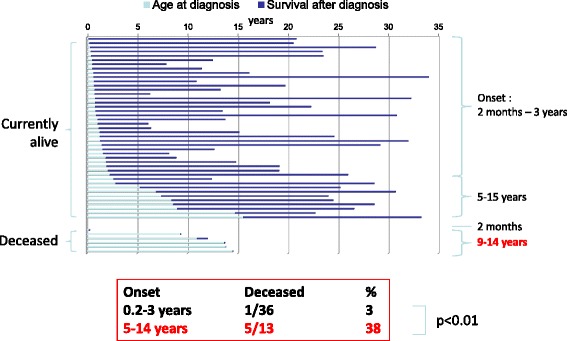
Age at diagnosis, length of follow-up, and mortality in the 1 mth-16 y group. A peak of mortality was noted approaching and during adolescence.

Mortality was much higher in the neonatal group (20/27 or 74% deceased at diagnosis or during follow-up) than in the late-onset forms (8/63 or 13% deceased overall, p < 0.0001; cf Figure 1). In fact, mortality reached 90% in the neonatal-onset group when the 6 patients diagnosed antenatally were removed from this group. As summarized in Table 1, in the neonatal group, 13 patients died early in the neonatal period; 3 died after postoperative liver transplantation at 3.5 years of age; 1 died of Reye syndrome at 4 years of age; and 3 died after infection with neurological signs and coma, and psychiatric signs in one patient, at ages 2, 6, and 7 years. To summarize, 60% of the cases with a neonatal presentation died at presentation, and 75% of the survivors subsequently died between 1 and 7 years of age. In the late-onset forms of the disease, 6 patients died at presentation during the first acute metabolic crisis between 9.3 and 55 years; 1 girl died at 12 years of age with a diagnosis of fibrolamellar hepatocellular carcinoma that was resistant to chemotherapy (her OTCD was diagnosed at age 11); and 1 girl died at 4 months of age after an infection that led to a hyperammonemic coma. This result indicates that for the late-onset form, deaths were rare during infancy, whereas a peak in mortality occurred at adolescence (9-14 years) in the 1 mth-16 y group (Figure 2; Fisher’s exact test, p < 0.01).

### Clinical presentation

The clinical presentation was homogenous in the neonatal-onset group, with lethargy, failure to thrive and vomiting that led to a hyperammonemic coma in 21 symptomatic patients. For the patients in the 1 mth-16 y group, the symptoms were quite variable (Table 2). The first symptoms were mostly neurological (49/52 patients) and included alterations in consciousness (31/52), global hypotonia (10/52), ataxia (2/52), and acute neurological regression (2/52). Three patients presented a sudden neurological deficit (hemiparesis) that was associated with seizures and fever referred to as stroke-like episodes at the ages of 6, 10 and 27 months. A pre-existing history of recurrent vomiting (3/3) and developmental delay (2/3) was retrospectively noted. Recurrent vomiting was frequently associated with the neurological symptoms (42/52 patients), whereas acute hepatic failure and/or Reye syndrome were less frequent (23%). Psychiatric symptoms were the primary presenting factors in 21% of the cases and involved abnormal behavior and confused states with hallucinations over time and disorientations in space. Nevertheless, in all of the cases, concomitant neurological or digestive symptoms were apparent, as detailed in the next section.

**Table 2:** **Clinical presentations of 90 patients with OTC deficiency**

**Initial symptoms**	**Neonatal-onset form**	**Late-onset form (1 m-16 y)**	**Adults (>16 y)**
	**(n = 27)**	**(n = 52)**	**(n = 11)**
Coma	21	14	4
Antenatal diagnosis (no symptoms)	6		
Neurological signs			
Alterations of consciousness		32	11
Global hypotonia		10	0
ataxia		2	0
acute regression		2	0
hemiparesis (« stroke-like »)		3	0
Digestive and Hepatologic signs			
recurrent vomiting		42	6
Reye’s syndrome		12	0
Psychiatric symptoms		11	4

All of the patients in the group > 16 y presented neurological signs at diagnosis (alteration of consciousness). Four of 11 patients presented with hyperammonemic coma, and 2 died recently at their first episode, including one as recently as 2011. Six of 11 patients had digestive signs (vomiting), but the psychiatric symptoms led to the diagnosis in 4/6 and again were consistently associated with neurological symptoms. A pre-existing history of chronic headaches (3/11) and repeated episodes of unexplained hepatitis (2/11) was retrospectively noted. Not surprisingly, 7/11 patients in the group > 16 y had an aversion to protein and spontaneously consumed a vegetarian diet.

### Decompensations

The number of decompensation episodes varied between the patients with the neonatal form who survived beyond the first week of life (14/27; actual mean age of 10.5 years for the 6 survivors in 2011) and those in the 1 mth-16 y group (actual mean age of 17 years for the patients who were alive in 2011). Three patients with a neonatal-onset had a score of 0 (patients with a prenatal diagnosis), seven patients had a score of 1 and three patients had a score of 2. The youngest patient was not included in the neurological and decompensation scores because he was 6 months old with normal development and had not yet experienced decompensate. The mean number of decompensations in this group was 6.2 (0-20). In the 1 mth-16 y group, 26 patients had a score of 0, 10 patients had a score of 1 and only 3 patients had a score of 2. The mean number of decompensations in this group was 2.5 (0-14). The difference was statistically significant between the two groups (Fisher’s exact test, p = 0.028). For patients in the group >16 y, the mean number of decompensations was 1.4 (0-3) (actual mean age of 44 years for the surviving patients in 2011). The survival rate might be related to the number of decompensations in the neonatal group, whereas in the 1 mth-16 y group, there was no relationship between the survival and frequency of decompensations (data not shown). Triggering factors for young patients or patients in the 1 mth-16 y group were primarily weaning from breastfeeding and/or dietary diversification (45%); infections were frequent (30%), and other triggers were less frequent: surgery (2 cases), sodium valproate (1 case), and vaccinations (2 cases). For patients > 16 y, 5/11 (45%) presented acute metabolic decompensation after an increase in protein intake during a meal. One woman had digestive symptoms with hyperammonemia post-partum.

### Neurological outcome

Five patients with a neonatal-onset had a median IQ = 90 (67-91) at ages 14 months, 16 months, 26 months, 5 years and 11 years. Three of the five patients are still alive at ages 3, 4 and 14 years. Among the surviving school-aged children, three had normal schooling and two had remedial schooling due to specific learning disabilities (dyslexia, deficits in executive function, attention deficit or hyperactivity).

In the 1 mth-16 y group, 20 patients between 2 and 12 years had a median IQ = 92 (55-103) (we considered the latest test for each patient). Among 38 patients in this group whose developmental outcome and schooling were precisely documented, 27 had normal schooling (71%), 5 had no schooling (13%) due to severe neurodevelopmental disabilities (severe epilepsy and mental retardation) and 6 had remedial schooling (16%). The two youngest patients (2 years old) were not yet documented because of their young age and were strictly normal. The mean neurological score for this group was 1.28 (n = 38) with 10 patients in group 2, whereas the mean neurological score in the neonatal group was 1.20 (n = 6) with only 1 patient in group 2. There were no statistically significant differences in the neurological scores between the neonatal-onset group and the 1 mth-16 y group. No significant correlation was detected between the neurological score and either the ammonia peak at diagnosis or the plasma glutamine concentration at diagnosis (data not shown).

Finally, among all of the adult patients at the time of the study, 7 were diagnosed during the neonatal period or childhood, and 9 were diagnosed in adulthood. All of the adults had good socio-professional and economic integration and seven had children, including one woman who had undergone liver transplantation at 6 years of age.

### Biochemical parameters at diagnosis

**Figure 3:**
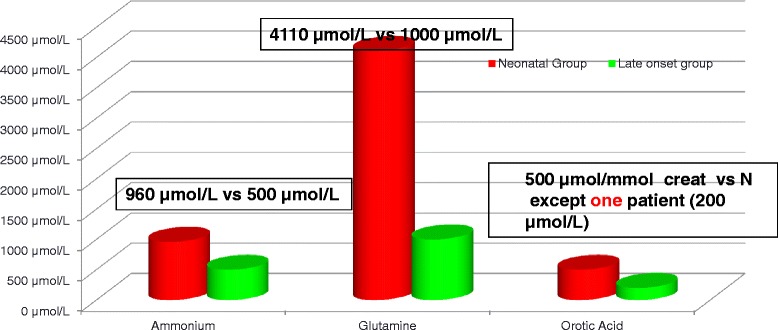
Comparison of biochemical parameters at diagnosis (plasma ammonemia, plasma glutamine and urinary orotic acid) in the neonatal group versus the late-onset groups (1 mth-16 y and >16 y groups).

**Figure 4:**
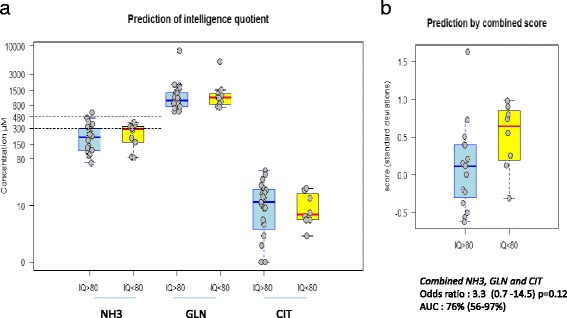
Initial plasma markers (plasma ammonemia, glutamine and citrulline) compared to the control group and cognitive outcome in the 1 mth-16 y group. Cognitive outcome was based on an IQ < 80 or >80, revealing extensive overlap of the values among patients with different outcomes. **a**: Individual markers shown separately (in μM). **b**: A combined score based on ammonia, glutamine and citrulline levels at diagnosis as a borderline predictor of IQ in the 1 mth-16 y group (see text).

The mean peak of ammonemia at diagnosis was much higher in the neonatal group after the exclusion of the patients who were diagnosed antenatally (mean value = 960 μmol/L and median value = 925 μmol/L) than in the pooled late-onset groups (mean value = 500 μmol/L and median value = 240 μmol/L). The plasma glutamine levels at diagnosis correlated with the ammonia levels and were much higher in the neonatal group (mean and median values = 4110 μmol/L) than in the late-onset groups (mean and median values = 1000 μmol/L) Figure 3. The degree of plasma citrulline depletion was more marked than that of plasma arginine in all of the patients (data not shown). The plasma concentration of citrulline was lower in the neonatal group (median value = 5 μmol/L and undetectable in 8 patients) than in the late-onset groups (median values = 15 μmol/L in patients in the 1 month – 16 year group and 14 μmol/L in patients in the greater than 16 year group). There was no correlation between the biochemical parameters at diagnosis (plasma ammonia, glutamine and citrulline) and the neurological outcome in the 1 m-16 y group (Figure 4a). Therefore, in patients with the late-onset form (1 mth-16 y), we attempted to combine three relevant biomarkers into a predictive score by regressing the plasma ammonia levels on the glutamine/citrulline ratio (intended as a better indicator of the glutamine response to ammonia rather than to other factors such as the fasting time). The resulting residuals can be rationalized as the among-patient variation of ammonemia relative to the glutamine levels, with higher values representing a reduced capacity to incorporate ammonemia into glutamine. The values for this score at diagnosis were borderline predictive of IQ, with an odds ratio of 3.3 (0.7 -14.5; p = 0.12) and area under the receiver operating characteristic curve of 76% (p < 0.05; Figure 4b).

**Figure 5:**
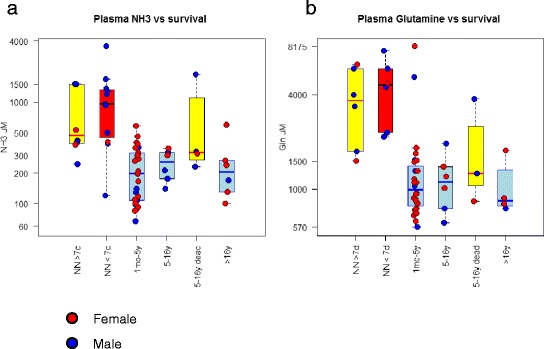
Biochemical markers in the different groups of patients with different clinical forms based on age at diagnosis as indicated. **a**: Plasma ammonia in μM. **b**: Plasma glutamine in μM. Red circles indicate female patients, and blue circles are male patients. The boxed color codes are approximate indications of the clinical severity (blue: moderate; yellow: severe; red: early severe).

We also investigated potential correlations between plasma markers at diagnosis and survival, and we found no differences in the ammonemia or glutamine levels between patients with a neonatal presentation that survived less vs those that survived more than 1 week (left-most columns in Figure 5a and 5b). The late-onset forms had reduced levels of glutamine and ammonemia, with potentially moderately higher levels in the group of patients who died during adolescence (Figure 5, yellow bar labeled as “5-15 y dead” compared to the pale blue bars).

The excretion of urinary orotic acid was very high at diagnosis in the neonatal as well as in the patients in the 1 mth-16 y group, with mean creatinine values of approximately 500 μmol/mmol. By contrast, the adult patients displayed normal urinary orotate excretion at the time of diagnosis (<6 μmol/mmol creatinine), excluding one patient (urinary orotic acid of 200 μmol/mmol creatinine).

**Figure 6:**
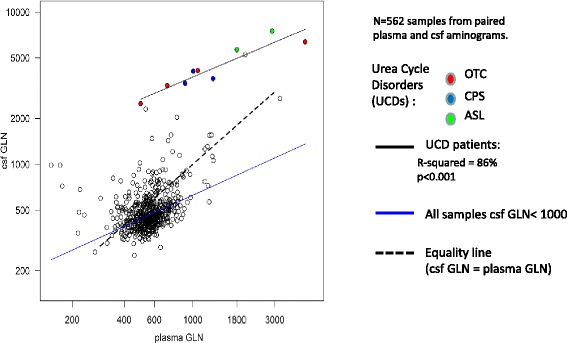
Biplot of CSF (Y-axis) vs plasma (X-axis) glutamine in paired samples from our hospital cohort (bilog transformation). Empty circles are the reference hospital population, and colored circles represent urea cycle disorders as indicated. The black solid line is the regression line for urea cycle disorder, the blue solid line is the regression line for the reference population (calculated based on samples with CSF glutamine levels less than 1000 μM), and the dotted line is the theoretical equality line between the CSF and plasma.

In 4 cases, we measured the glutamine level in the cerebrospinal fluid (CSF) concomitant with the plasma glutamine. Four patients (6 months, 6 months, 4 years, and 19 years old) had very high CSF glutamine (2500-7482 μmol/L) levels compared to their plasma glutamine levels (500-4500 μmol/L) during acute metabolic decompensations in which ammonemia was subnormal or moderately high, suggesting that neurological symptoms and cerebral edema were caused by an increase in brain glutamine. In a fifth patient aged 40 years, CSF glutamine had also been collected a few days after a metabolic decompensation that occurred during follow-up (1857 μM), but no plasma was collected at that time. Figure 6 compares the 4 cases with paired plasma/CSF glutamine determinations to the paired samples from other urea cycle defects and to a reference hospital cohort of 562 paired CSF plasma samples of non-UCD patients. We found that the level of CSF glutamine was systematically much higher in OTCD and other UCD relative to the reference population (as summarized by the black vs the blue regression lines in Figure 6). Thus, compared to plasma glutamine, CSF glutamine may represent an additional marker for the diagnosis of defects in the urea cycle. Nevertheless, among patients with OTCD and urea cycle disorders, CSF glutamine varied linearly with plasma glutamine (upper regression line in Figure 6; r-squared = 0.86). Thus, CSF glutamine appears to be as informative as plasma glutamine during follow-up. However, because of the small sample size, we cannot exclude the possibility that some of the patients with UCD may show normal CSF glutamine levels at presentation.

### Biochemical parameters of heterozygous females

In a heterozygous OTCD female, which is often but not always identified among the relatives of probands, we found a broad spectrum of symptoms ranging from protein aversion and behavioral disabilities, making it difficult to distinguish between symptomatic and asymptomatic carriers. Most of these heterozygous female patients (approximately 80% in the literature) are apparently asymptomatic and present a normal biochemical workup (notably, ammonemia and plasma glutamine). In our cohort, among the asymptomatic carrier females, only 2/25 had abnormal peak urinary orotate (>10 μmol/mmol of creatinine), and 7/18 had a positive protein loading test with increased acid orotic excretion in the urine and/or high ammonemia in the plasma. These loading tests and the allopurinol test are not used for the diagnosis of asymptomatic females due to the lack of sensibility, and genetic analysis is currently the screening tool used to diagnose female carriers. Because these asymptomatic carrier females were healthy, exhibiting no decompensation during environmental-related stress episodes (fever, gastroenteritis, surgeries, deliveries) and normal biochemical data under baseline conditions, no treatment was proposed. However, an emergency certificate was provided to manage any symptoms related to acute metabolic stress situations.

### Biochemical and therapeutic follow up

Regarding the long-term biochemical follow up, ammonia levels were normal in the absence of episodes of decompensation (<50 μmol/L). We analyzed plasma glutamine and urinary orotic acid independently of acute metabolic decompensations for each patient with a follow-up between 3 and 14 years after diagnosis. The mean and median glutamine values were approximately 800 μmol/L (548-1123) for all of the patients. The urinary orotic acid measurements revealed a large range of inter- and intra-individual variability, with mean and median values of 20,6 μmol/mmol (2,5-115) and 10 μmol/mmol of creatinine, respectively. Furthermore, the levels of plasma glutamine and orotic acid did not correlate within the same patient.

Concerning the long-term management, surviving patients in the neonatal-onset group after the neonatal period were treated with a low protein diet (mean 1 g protein/kg/day at one year of age for 8 patients, three years of age for 4 patients and six years of age for 4 patients). Three patients required nasogastric enteral feeding. In the 1 mth-16 y group, the mean total protein intake was 1.3 g protein/kg/day at one year and then diminished to 1.1 g protein/kg/day at three years and 0.9 g protein/kg/day at six years. Nasogastric enteral feeding was required for 10 patients (20%) due to anorexia (8/10) and to neurological sequelae with deglutition disorder (2/10). For patients > 16 y, a low protein diet was not systematically implemented (i.e., 75% of the patients); the diet was moderate with spontaneous protein restriction to approx. 35 to 50 g protein/day.

Arginine supplementation was given without citrulline in 42% of the neonatal patients and 30% of the late-onset groups, citrulline alone in none of the neonatal patients and 28% of late-onset groups and both arginine and citrulline in 70% of the neonatal patients and 23% of the late-onset groups. Arginine and citrulline were combined with the ammonia scavengers oral sodium benzoate for only 50% of the patients in neonatal group and 38% of the patients in late-onset groups; sodium phenylbutyrate was never used alone in late-onset groups and in only 10% of the neonatal patients. These treatments were used in association in 40% of the patients in the neonatal group and 23% of the patients in the 1 mth-16 y group (not in patients >16 y).

Among patients with the neonatal form of the disease, two boys and one girl underwent liver transplantation at three and a half years of age but died during the immediate postoperative period due to surgical complications (acute transplant rejection, vascular complications and acute tacrolimus toxicity). In the late-onset group, two girls underwent orthotopic liver transplantation from a cadaveric donor at three and a half and six years of age. The indications for liver transplantation were recurrent acute episodes of hyperammonemic decompensation despite aggressive dietary and therapeutic treatment. Those who survived beyond the postoperative period are alive at age 19 and 25 years at the end of the study.

Among the adult patients, only one female was pregnant. The delivery was medically assisted (intravenous sodium benzoate, arginine, glucose and lipids) with no complications. Her daughter, an affected infant, is now treated in the unit.

### OTC activity and mutation analysis

The diagnosis was confirmed by the measurement of hepatic (16 patients) or digestive (21 patients) OTC enzyme activity and/or by mutation analysis (68 patients investigated). OTC enzyme activity was undetectable in the neonatal-onset group (8 patients). In the late-onset groups, 29 biopsies (13 liver biopsies and 16 intestinal biopsies) revealed a mean value of residual OTC enzyme activity of 11% (2-25%). Molecular diagnosis by direct sequencing of the ten exons identified a deleterious mutation in 60/68 patients (88%), with 43 different mutations (Table 3). In the neonatal group, 63% (14/22) of the mutations were single-base substitutions, whereas the remaining mutations were complete deletions (18%, 4/22) or mutations affecting consensus splicing sites (18%, 4/22). No prevalent mutations were noted in this group. In the late-onset forms, single base substitutions were also the most frequent (73%, 27/37), and the remaining mutations were large and small deletions (19%, 7/37) and splice site mutations (8%, 3/37). One mutation has been described previously to confer a late-onset type of presentation [14]: c.119 G>A (single base substitution: pArg40His in exon 2 or R40H). This mutation was recurrent and was detected in 13% of the 1 mth-16 y group (4/30) and 57% of the group >16 y (4/7). Thus, the mutations in the neonatal and late-onset groups were distinct.

**Table 3:** **Deleterious mutations in OTC genes**

	**Neonatal-onset form**	**Group (1 mth-16 y)**	**Group >16 y**	**Carriers**
Deleterious mutations	22	30	7	52
				10 F / 42 M
• Single base substitution	14	21	6	
• Small deletions	0	3	1	
• Large deletions	0	3	0	
• Complete deletions	4	0	0	
• Splice site mutations	4	3	0	
Recurrent mutations	R40H :0	R40H :4	R40H :4	R40H :20
	T178M :1 M	T178M :2 F	T178M :1 F	T178M :6 F
De novo mutations	8	10	4	
X-linked mutations	14	20	3	
Mutations transmitted by father	0	0	0	

M: male; F: female. NB: two families with asymptomatic fathers who transmitted R40H mutations to their asymptomatic daughters.

Half of the mutations were inherited in males in the neonatal group and in the 1 mth-16 y group. In females, mutations were inherited in 60% of the neonatal group and in 24% of the 1 mth-16 y group. In all of the patients, 1/3 mutations were de novo and 2/3 mutations were X-linked inherited mutations without differences between the groups.

## Discussion

Here, we report a series of 90 patients with OTCD, of which 30% had a neonatal and 70% had a late-onset form (revealed at ages 1 mth-16 y or >16 y). The gender distribution was not equivalent between boys and girls, as expected from an X-linked disorder, and therefore, 81% of the neonatal cases involved boys, whereas girls presented more frequently during childhood [15]. The survival rate was 30% in the neonatal forms, which is comparable to a previous study [15], and decreased to 10% with the removal of the patients who were diagnosed prenatally. However, only eight neonates in our series were born after the year 2000 as a consequence of progress in intensive medical therapies along with improved hemofiltration [7] and liver transplantation. However, presymptomatic therapy may improve neonatal survival (100% vs 40%, p = 0.06) and long-term survival (50-75% vs 10%, p = 0.11). It is also important to consider that some adults still die during their first metabolic coma because of a delayed diagnosis [16], thus emphasizing that adult intensive care unit physicians should have a greater awareness of the presentations of late-onset forms of inherited metabolic diseases.

### Variable clinical presentation: is OTC deficiency still underdiagnosed?

The OTCD phenotype ranges from neonatal or adult death (hyperammonemic coma) to various hepatic, psychiatric and neurological symptoms including gastrointestinal dysmobility. Several adults experienced their first symptoms as late as 60 years of age [17], but they were likely to represent only a fraction of a larger pool of affected adults. The most suggestive symptoms are neurological (lethargy, ataxia, hypotonia, confusion, headache) associated with recurrent vomiting (gastrointestinal dysmobility, a neurological symptom). Although rare in our series, focal neurological sign such as hemiplegia and stroke-like episodes can also occur [5], which may be due to alterations in vascular endothelial wall integrity and in cerebral perfusion [18]. Psychiatric symptoms were relatively common in our cohort but were always associated with other neurologic or digestive signs. Nevertheless, psychiatric symptoms were the leading cause of consultation in several cases, suggesting that the diagnosis of OTCD may still be underdiagnosed. Overall, we advocate that irrespective of age, any individual with an encephalopathic illness, psychiatric symptoms, stroke-like episodes or suspicion of exogenous intoxication should undergo prompt blood ammonia estimation due to the possibility of a diagnosis of OTCD [19].

### Neurological outcome of surviving patients

Concerning the neurological outcome in surviving patients, we obtained satisfying results for the IQ/DQ tests and neurological score although they were not conducted at the same age for all patients (IQ 90 to 92 for tested patients, normal schooling for most patients and normal socio-professional insertion for adults). Like others before us [20,21], we found that prospectively treated children (antenatal diagnosis) had a better neurological outcome because the management of the first acute coma is crucial.

We did not perform neurocognitive testing of the apparently asymptomatic female carriers, although some of them appeared to be emotionally fragile and emotive. Previous studies have identified some degree of neurological disability. In Gyato’s study [22], comprehensive neuropsychological testing was conducted in 10 mildly symptomatic and 9 asymptomatic heterozygous women. Among the group, these women had average IQ scores and displayed specific neuropsychological phenotypes with significant strengths in verbal intelligence, verbal learning, verbal memory and reading. In contrast, they scored significantly lower than the normative sample for nonverbal and fine motor functions. The asymptomatic subgroup outperformed the symptomatic subgroup in all of the tested domains.

At the other end of the spectrum, we identified 10 asymptomatic boys among the relatives of the probands who could have mimicked dominant inheritance with incomplete penetrance.

### Biochemical parameters

Significant differences were found for the plasma ammonia concentration at diagnosis, with mean values of approximately 960 μmol/L in the neonatal group and approximately 250 μmol/L in the late-onset groups, which is consistent with previous studies [14]. There are very little data in the literature concerning other biochemical markers. Regarding plasma glutamine, Tuchman’s study [23] showed a mean value of 859 μmol/L, which was much lower than the value obtained in our study (mean value of 4110 μmol/L in the neonatal-onset group versus 1000-1500 μmol/L in the late-onset forms). In addition, the mean values for plasma citrulline were much lower than in Tuchman’s cohort (15 μmol/L versus 29 μmol/L). These differences were related to the smaller proportion of neonatal patients and the large number of asymptomatic females with normal values in Tuchman’s group. Only the arginine concentration showed similar values in the two cohorts.

In other studies [7,24] as well as in ours, there was a significant correlation between survival and the peak level of ammonium. In our cohort, survival was significantly improved when peak ammonium levels were less than 500 μmol per liter compared to more than 1000 μmol per liter (*p < 0.001*, Fisher’s exact test). Among the eleven patients (10 neonates and 1 infant) with peak ammonium levels greater than 1000 μmol/L, 10 died within the few days following the hyperammonemic episode (survival: 9%), whereas among the 46 patients with peak ammonium levels less than 500 μmol/L, survival was significantly improved (survival: 90%).

In the 25-year retrospective cohort of Enns [7], which included 299 patients with urea cycle disorders with episodes of hyperammonemia, the overall survival was 84% with a combination of intravenous phenylacetate and sodium benzoate therapy (Ammonul®). Nevertheless, patients who were younger than 30 days of age with a peak of ammonium level above 1000 μmol/L were the least likely to survive a hyperammonemic episode (38%) despite the use of drugs and hemodialysis. By contrast, there were no significant correlations between the plasma glutamine concentration at diagnosis and the neurological outcome in our cohort. Nevertheless, a combined “score” reflecting excess plasma ammonia levels that were normalized to glutamine as measured at diagnosis (see Results section above) was a borderline predictor of low IQ in late-onset OTCD (OR: 3.3; p = 0.12; and AUC: 76%, p < 0.05). This score was meant to measure the ammonia-scavenging capacity of glutamine: the greater such a “normalized difference” between ammonia and glutamine, the lower was the inferred ammonia scavenging capacity. It will be interesting to further evaluate whether this score or any other parameter combining ammonia and amino acid levels may improve prognosis or follow-up.

The presence of glutamine in plasma does not fully reflect the concentration of glutamine in CSF [25] and in brain tissue. Ammonia enters the brain via diffusion from the blood or cerebrospinal fluid, or it is formed in situ from the metabolism of endogenous nitrogen-containing substances. In the brain, glutamine synthetase confined to astrocytes increases the formation of glutamine from ammonia, generating an osmotic stress that compromises astrocyte function and morphology and leads to neuronal dysfunction (« the osmotic gliopathy theory »).

We found that the levels of CSF glutamine were systematically elevated in all 4 tested patients with OTCD as well as in patients with other UCD compared to the complete hospital cohort, and this finding contrasted with plasma values that largely overlapped with the normal/reference values (Y vs X axis in Figure 6). Otherwise, the CSF and plasma glutamine values were highly correlated in these patients (r-squared of 86%, Figure 6). Thus, it is unclear whether CSF glutamine provides added value compared to plasma glutamine in patients with known UCD, whereas it likely provides information for diagnostic purposes. Because glutamine tends to increase as a first-line buffer against ammonia accumulation, it is an important early marker of UCD decompensation. Nevertheless, the normalization of glutamine to other amino acids, notably citrulline (cf. Figure 4), may be preferable to control for confounders such as the sensitivity of amino acid levels to the fasting status.

### Therapeutic strategies

The standard therapy used for acute hyperammonemic episodes includes ammonia scavengers (intravenous or oral), arginine and citrulline supplementation, and hemodialysis. However, no consensus or established protocol is currently available, and thus, our data reflect our own practices. Moreover, our practices have changed over time: the neonatal patients are now usually treated with both arginine and citrulline and with ammonia scavengers including the combination of intravenous sodium phenylacetate and sodium benzoate, according to the European protocol to which we have contributed [7]. In 2013-2014, the three neonates we cared for at birth for OTCD and who were not included in this report, were successfully resuscitated by medical management (data not shown, see below).

In addition, liver transplantation is an option for the management of patients with severe urea cycle disorders, in particular patients who present neonatally or those with poor metabolic control and recurrent episodes of decompensation despite optimal medical treatment. In our series, three deaths among five patients who underwent liver transplantation occurred during the early postoperative period due to technical problems (acute transplant rejection, vascular complications and acute tacrolimus toxicity). In addition, we recently changed other practices (surgery, intensive care, anesthesia), which may explain the increased rate of success. For example, among the three neonates who were recently born and not included in this series, two successfully underwent liver transplantation at ages 9 and 6 months. In a review of the current role of transplantation for the treatment of urea cycle disorders [26], the overall cumulative patient survival rate was found to be more than 90% at 5 years (6 deaths among 51 patients). Among other groups, experience in Japan shows that systematic aggressive liver transplantation for OTCD patients diagnosed at birth since 1995 has improved survival from 10% to currently 90% [27]. Although the reported success rate may be slightly inflated by the inclusion of patients who benefited from a prenatal diagnosis and thus preventive therapy at birth, these studies show that liver transplantation has dramatically improved the survival of neonatal-onset OTCD patients. In France, based on these results, the indications for liver transplantation are currently being extended. Nevertheless, practitioners are still concerned about the quality of life of the transplanted OTCD patients. Moreover, limited resources, including the supply of donor livers, may restrict liver transplantation options. An alternative therapy for urea cycle disorders may be hepatocyte transplantation. However, all of the biochemical and clinical corrections described to date appear to have been transient, and hepatocyte transplantation was followed by liver transplantation [28]. Finally, hepatocyte transplantation could be used in conjunction with auxiliary partial orthotopic transplantation as an effective treatment for severe OTCD patients and likely improve neurodevelopmental outcomes [29].

### Diagnostic confirmation

Molecular analysis is now regarded as the reference test for the identification of private mutations within families. This technique is not invasive, in contrast to the liver or intestinal biopsies required for enzyme assays, and patients with mild disease and asymptomatic relatives of patients are being diagnosed with increasing frequency [14]. In the literature, only approximately 50 to 80% of patients with OTCD have an identifiable point mutation based on sequencing analysis [14,30]. In our cohort, we identified 87% of the deleterious mutations. Only 9/68 patients had negative molecular results but a reduced OTC enzyme activity. Most mutations in OTC genes were private and specific to a family, and more than 340 mutations are known to cause OTC deficiency [30]. The most frequent mutations in our cohort were single base substitutions (70%) as described previously [14], and mutations have been found in all of the exons and introns of the OTC gene. Among single nucleotide substitutions, substitutions of G to A and C to T were much more frequent than any other substitution, as observed in the « recurrent » R40H mutation (c.119 G>A) and the T178M mutation (c.533C>T). This finding can be explained by the mutagenic CpG dinucleotide (methylation-induced deamination). Large deletions and deletions of the entire gene were more frequent (12%) than in the others studies [14,30] (approximately 5%), but multiplex ligation-dependent probe amplification (MLPA) analysis was systematically performed in our series. Thus, deletions should be searched by array comparative genomic hybridization (CGH array) or MLPA in the case of negative OTC sequencing in patients with symptoms of the disease [13,31]. OTC deletions can be of various sizes (intragenic deletions of one or more exons or deletions of the entire gene) [32]. It is important to note that the 4 complete deletions of the OTC gene were observed in the neonatal presentation. Mutations affecting the consensus splice site represented approximatively 12% of the total mutations, which is consistent with the McCullough report. In the literature, consensus splice site mutations appear to confer a severe neonatal-onset phenotype. In our series, two splice site mutations affecting consensus intron sequences (IVS7+3A>G and IVS5a A>G) were previously described to confer a severe neonatal phenotype and were identified in two females herein with a late-onset presentation. The remaining 5 to 10% of the patients without a mutation (neither large deletions nor point mutations) could carry mutations in regulatory domains (promoter or enhancer) or intronic regions of the OTC gene, or they could have mutations in other genes that confer a phenocopy of OTCD.

Several mutations found in asymptomatic adults or adults with a mild phenotype were also detected in children with a severe phenotype. This finding suggests that OTC deficiency is a highly pleomorphic, semidominantly inherited disorder in which other genetic and environmental factors such as catabolic events play an important role. Despite a better understanding of the normal function and assembly of OTC, it remains difficult in some cases to predict what phenotype to expect for a particular mutation. Interestingly, OTCD was initially assumed to be transmitted by females only as an X-linked disorder. However, only half of the mutations (for example T178M, R92C and G39C) were inherited in males in the neonatal group. We identified adult males with partial OTCD, some of which were asymptomatic, but they transmitted their mutation to their asymptomatic daughters, such as R40H and A208T mutations. Overall, it is very important to identify the mutation carried by the affected patient and then to screen the entire family (carrier testing and prenatal diagnosis) to identify asymptomatic relatives. Preventive measures are important for relatives because death can still also be associated with late presentations of the disease. Thus, genetic screening is crucial for the prevention of acute decompensation in relatives.

## Conclusion

Despite medical progress and prompt diagnosis, morbidity and mortality have remained unsatisfactorily high in the neonatal group of our cohort, but by analogy with other countries, mortality should decrease as hepatic transplantation becomes a more consensual indication for this disease in France. In the adult group, death may still occur during the first episode of decompensation due to a delayed diagnosis. Thus, we strongly emphasize that critical care clinicians be aware of the crucial need for early diagnosis in patients with adult-onset OTC. Although survival differed between clinical forms and correlated with lower ammonia and glutamine levels, the neurological outcomes of the survivors did not correlate with hyperammonemia or plasma glutamine levels at diagnosis. Mutations were only partly correlated with the clinical presentation, emphasizing the influence of other genetic or environmental metabolic factors on the life history of this semidominantly inherited disease.

## Abbreviations

OTC: Ornithine Transcarbamylase
UCD: Urea cycle disorder
DQ: Developmental quotient
IQ: Intellectual quotient
MLPA: Multiplex Ligation-dependent Probe Amplification
CSF: Cerebrospinal Fluid
